# Intellectual Functioning in Children with Congenital Heart Defects Treated with Surgery or by Catheter Interventions

**DOI:** 10.3389/fped.2016.00113

**Published:** 2016-11-17

**Authors:** Carmen Ryberg, Jan Sunnegårdh, Maria Thorson, Malin Broberg

**Affiliations:** ^1^Psychology Unit, Department of Pediatric Cardiology, The Queen Silvia’s Childrens Hospital, Sahlgrenska University Hospital, Gothenburg, Sweden; ^2^Department of Pediatric Cardiology, The Queen Silvia’s Childrens Hospital, Sahlgrenska University Hospital, Gothenburg, Sweden; ^3^Department of Psychology, University of Gothenburg, Gothenburg, Sweden

**Keywords:** intellectual functioning, neurodevelopment, congenital heart defects, cardiac treatment by surgery or by catheter interventions

## Abstract

**Background:**

Studies suggest that children with congenital heart defects (CHD) are at risk for adverse intellectual functioning. However, factors related to lower intellectual functioning in this group are largely unknown. This study describes intellectual functioning in children with CHD in relation to severity of the heart defect, the child’s age, and the socioeconomic status of the family (SES).

**Methods:**

Two hundred twenty-eight children treated with surgery or by catheter technique were tested using the Wechsler intelligence scales to determine full scale IQ (FSIQ). FSIQ was then analyzed in relation to age (3-, 5-, 9-, and 15-year olds), severity of the diagnosis (mild, moderate, and severe), and SES (low, medium, and high). The median age was 70 months (5.8 years) with a range of 162 months [30 months (2.5 years) to 192 months (16.0 years)].

**Results:**

The total mean score on FSIQ was 100.8 (SD = 14.5). Children with severe CHD had significantly lower FSIQ than children with mild and moderate CHD, and 9- and 15-year olds had significantly lower FSIQ compared to the 3-year olds. Children from families with low SES had significantly lower FSIQ than children from medium SES and high SES families. No interaction between severity of diagnosis, age, and SES was found for FSIQ.

**Conclusion:**

Eighty-three percent of the children with CHD performed at or above average with respect to FSIQ. SES and severity of diagnosis had significant main effects on FSIQ. These factors should be considered when planning interventions and follow-up programs for children with CHD.

## Introduction

Because children with congenital heart defects (CHD) now live longer due to advances in surgical and catheter techniques, their neurodevelopment, including intellectual functioning, has become a major area of concern (1, 2), attracting both clinical and research interest (3).

Severity of the heart diagnosis in children with CHD has been suggested as an important predictor for intellectual functioning: the more severe the heart diagnosis, the higher the risk for lower intellectual functioning (4) and poorer academic performance (5). Results, however, are not consistent. A study comparing univentricular and biventricular patients showed no significant group differences with respect to intellectual functioning; that is, both groups scored within the norms. However, when assessing specific neuropsychological functions within the domains of attention and executive and sensorimotor functioning, only the univentricular patients had lower scores compared to the matched controls (6).

The use of wide age ranges when measuring intellectual functioning in children with CHD have made it difficult to compare studies (5). A meta-analysis described a significant relation between older age and better cognitive functioning. Further analysis disclosed that when excluding patients with the more severe diagnosis, the age effect disappeared, implying that age effects were observed because patients with the more severe diagnosis were often tested at an earlier age (5). Some studies [e.g., Oates et al. (7)], however, have not found correlations between age and intellectual functioning (8).

The relation between SES and intellectual functioning is well investigated in the normal population (9–13) as well as in children with CHD (14–16). Intellectual outcomes in children with CHD are often shown to be related to maternal education (17), comorbidity (i.e., velocardiofacial syndrome), and low SES (18, 19). In a study of cardiac arrest in children with heart disease, higher SES correlated with higher intellectual functioning, lower levels of behavioral problems, and lower levels of parental stress (20). In studies done in healthy populations, intellectual functioning is positively related to SES (21).

Scientific knowledge on neurodevelopment in children with CHD, and specifically, intellectual functioning, is still incomplete. Previous studies have been limited to small groups of patients (22, 23) with a specific diagnosis (17, 24–26), have been overly restricted, specific, or unclear with respect to age groups (27–29), and have been limited to specific surgical procedures (26, 30). Using the intelligence quotient (FSIQ) score, the aim of the present study was to investigate intellectual functioning in children with CHD treated with surgery or by catheter techniques and to investigate if intellectual functioning was related to severity of the heart diagnosis, child age, or socioeconomic status of the families. The following hypotheses were tested:

1. Children with severe CHD have lower intellectual functioning than children with mild and moderate CHD,
2. Older children have lower intellectual functioning than younger children with CHD,
3. Children with CHD living in families with low SES have lower intellectual functioning than children in families with higher SES,
4. There is an interaction effect of severity of diagnosis and SES: children with low SES and severe CHD have lower intellectual functioning compared to children with high SES and severe CHD.

## Materials and Methods

### Participants

**Figure 1:**
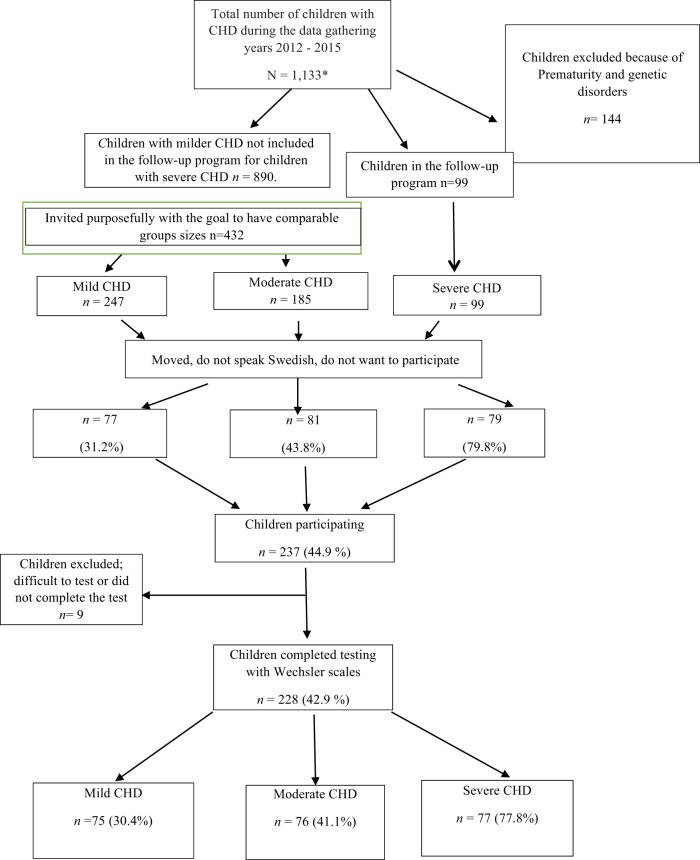
**Study population**. *Children treated with surgery or by catheter interventions for CHD.

Participants were tested over a 7-year period (2008–2015). In the beginning, only children with severe CHD living in the Gothenburg area were included. As time and financial resources allowed, children with severe CHD were recruited from the whole region of Västra Götaland. Later, children with milder CHD (a larger population) were recruited to obtain comparison groups of comparable sizes. Using the medical records of children living in the Västra Götaland Region (VGR), we know that 1,133 children were treated with surgery or catheter interventions for CHD at Queen Silvia Children’s Hospital in Gothenburg, Sweden during the data collection period. Children with chromosomal defects and disabilities known to influence intellectual functioning (*N* = 144) were excluded. The invited families were required to speak, read, and write Swedish and to provide a signed consent. All eligible children with severe CHD (*N* = 99) and 432 (of 890) children with milder CHD were invited. We were unable to locate some patients, and some patients did not want to participate for different reasons (e.g., lack of time, not wanting to worry the child who had a mild CHD and were unaware of any difficulties, and did not speak Swedish). In total, 531 patients were invited (Figure 1); of these, 237 children and their families (44.9%) agreed to participate in the study. Participation rate was higher in the severe group than in the milder groups. All children met with a clinical psychologist at their local hospital for a psychological evaluation.

Four hospitals participated in the gathering of data: The Queen Silvia Children’s Hospital, North Älvsborg County Hospital, Southern Älvsborg Hospital, and Skaraborg Hospital Skövde; most of the testing was done by the same psychologist, however, the groups of psychologists testing the children met regularly to assure test reliability. Nine of the tested children were not included in the analyses: two 3-year olds were unable to complete the tests; two 5-year olds had unreliable test results (one was given a neuropsychiatric diagnosis); and one 15-year old refused to finish the testing. Four 9-year olds were not tested with the Wechsler scales. As a result, 228 children (42.9% of those invited) completed testing with Wechsler scales at age 3, 5, 9, or 15 years (Table 1) and are included in the analyses; 111 (48.7%) were girls and 117 (51.3%) boys. Approval from the ethics committee in Gothenburg, Sweden was obtained on September 20, 2011 (ref. no. 391–11).

**Table 1:** **Participants grouped by the severity of their cardiac diagnosis and the targeted age for assessment**.

CHD	AGE				
	3 years	5 years	9 years	15 years	*N* (%)
	Min. 2.5 years, max 3.8 years	Min. 4.5 years, max 6.0 years	Min. 7.6 years, max 10.4 years	Min. 13.9 years, max 16.0 years	
Mild	19	17	19	18	73 (32.0)
Moderate	20	17	24	17	78 (34.2)
Severe	26	19	18	14	77 (33.8)
*N* (%)	65 (28.5)	53 (23.2)	61 (26.8)	49 (21.5)	228 (100)

*111 children (48.7%) were girls and 117 children (51.3%) were boys*.

### Intellectual Assessment

The Swedish versions of the Wechsler Scales of Intelligence (31, 32) were used to assess general intellectual functioning. The Wechsler Preschool and Primary Scale of Intelligence – third edition (WPPSI–III Swedish version) was used to test the 3- and 5-year-old children, and the Wechsler Intelligence Scale for Children – fourth edition (WISC–IV Swedish version) was used to test the 9- and 15-year-old children. Only core subtests were used to compute the full scale IQ (FSIQ). Analysis of verbal IQ (VIQ) and performance IQ (PIQ) did not show significant differences, so we report only FSIQ since it is the most robust measure of intellectual functioning. The children assessed with the WPPSI–III in the 2:6–3:11 age band were administered only five subtests: receptive vocabulary, block design, information, object assembly, and picture naming. For the children in the 4:0–7:3 age band, eight subtests were administered: information, vocabulary, word reasoning, block design, matrix reasoning, picture concepts, coding and symbol search. The children assessed with the WISC–IV, designed for children of 6:0–16:11 age, were assessed using 10 core tests: Similarities, vocabulary, comprehension, block design, matrix reasoning, digit span, coding and symbol search. The Swedish versions of the WPPSI–III and the WISC–IV are based on UK standardization data. Data have shown that previous British and Swedish versions of the Wechsler scales are comparable (31) as both measure similar constructs and their norms are highly consistent (33).

The Wechsler scales of IQ are based on the normal distribution curve with a mean score of 100 (SD 15); 68% of children in a population are expected to have an IQ score between 85 (−1 SD below the mean) and 115 (+1 SD above the mean), 14% are expected to have an IQ score between 116 and 130 (+2 SD), 14% are expected to have an IQ score between 84 and 70 (−2 SD), 2% are expected to have an IQ score between 131 and 145 (+3 SD), and 2% are expected to have an IQ score between 69 and 55 (−3 SD).

### Cardiac Diagnosis and Severity

The children in the study had a wide range of cardiac diagnoses and were divided into three groups. Recruitment of children with moderate and mild CHD was done with the aim of having approximately equal numbers of children as in the severe group (see Table 1 for distribution of these groups). The three severity groups were based on the risk the child had for further complications and their cardiac diagnosis: (1) the mild group – children usually treated once with surgery or by catheter intervention with little risk for further complications and who were, in most cases, no longer followed up, e.g., atrial septal defect, ventricular septal defect, persistent ductus arteriosus, isolated coarctation of the aorta, and pulmonary stenosis; (2) the moderate group – children who had been treated with surgery and/or catheter intervention and were followed up regularly since there were risks for further complications, e.g., transposition of the great arteries, tetralogy of Fallot, complete AV defect, total anomalous pulmonary venous drainage, and aortic stenosis; and (3) the severe group – children with complex heart defects for whom long-term prognosis was uncertain and serious complications were not uncommon, e.g., univentricular heart lesions, pulmonary atresia with ventricular septal defect and major aortopulmonary collaterals, and heart transplantation.

### Age

Testing was performed between 2008 and 2015. Families of children who met the inclusion criteria were invited when children were at the right age for testing. Children were tested as toddlers (3 years), pre-schoolers (5 years), school aged (9 years), or teenagers (15 years) (±6 months from their birthday), recruitment of patients from the moderate and mild CHD-group was done purposefully to get equal number of children in each age group to match the severe group. Ages for data gathering were chosen to the match the follow-up program for children with CHD at Queen Silvia Children’s Hospital in Gothenburg.

### Socioeconomic Status

The Hollingshead “Four Factor Index of Social Status” was used to determine socioeconomic status index (SES) for each family. This index weighs education, occupation, and employment status to determine a composite score of social status.^1^ From our sample of 228 children, 366 parents (202 mothers and 164 fathers) answered the questionnaire. In 17 families, none of the parents provided data on SES and for 154 families, both parents answered the questionnaire. When both parents provided data, we followed the Hollingshead manual’s requirements and divided the sum of their scores by two to create a SES index for the family (see text footnote 1). Based on a *Z*-transformation, three SES groups were formed: low (−1 SD), medium (±1 SD), and high (+1 SD). The scores on the index ranged from 3 to 66; the mean score for our sample was 43.6 (SD = 12.5), indicating that, on average, our patients came from medium to high SES families. Previous studies have shown an average SES of 37.0 (SD = 11.7) in the Swedish population (13).

### Statistical Analysis

SPSS 20.0 was used for the statistical analyses. The dependent variable – FSIQ – was normally distributed and therefore parametric tests were used. For between-group comparisons of intellectual functioning (FSIQ) in relation to age (3-, 5-, 9-, and 15-year olds), severity levels (mild, moderate, and severe) and SES (low, medium, and high), we used analysis of variance (ANOVA) with eta-square to analyze effect size, interpreting 0.02 as small, 0.13 as medium, and 0.26 as large effect size (34). For the *post hoc* tests, Bonferroni was used. All variables were check for normality. For non-parametric statistics, we used cross tabs and chi-square test (*p* ≤ 0.05 statistically significant).

## Results

### Intellectual Functioning

**Figure 2:**
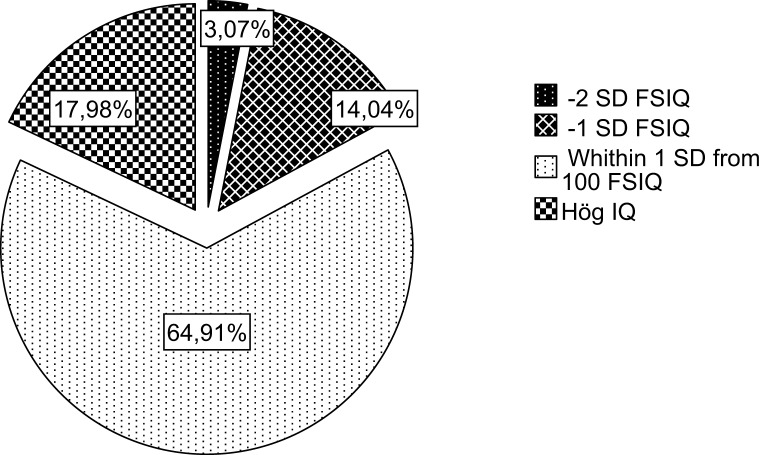
**Proportion of children performing in the normal range (±1 SD) and over (+1 and SD) and under (−1 and −2 SD)**.

**Figure 3:**
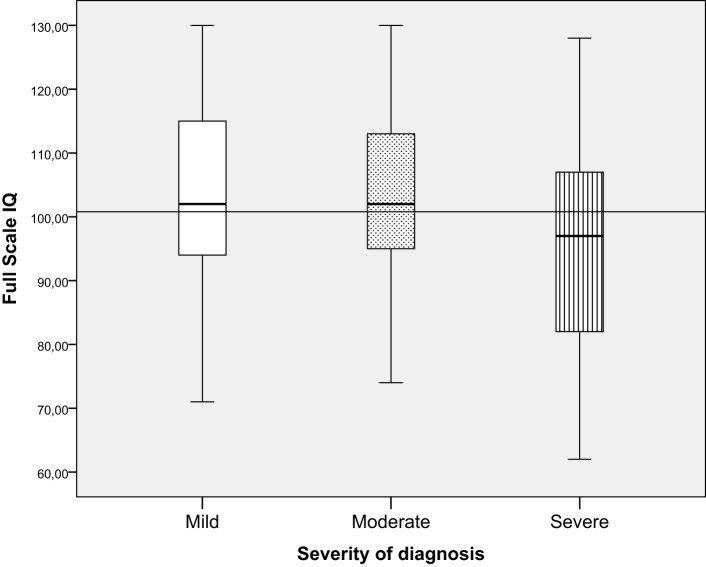
**FSIQ in relation to severity of diagnosis**.

**Figure 4:**
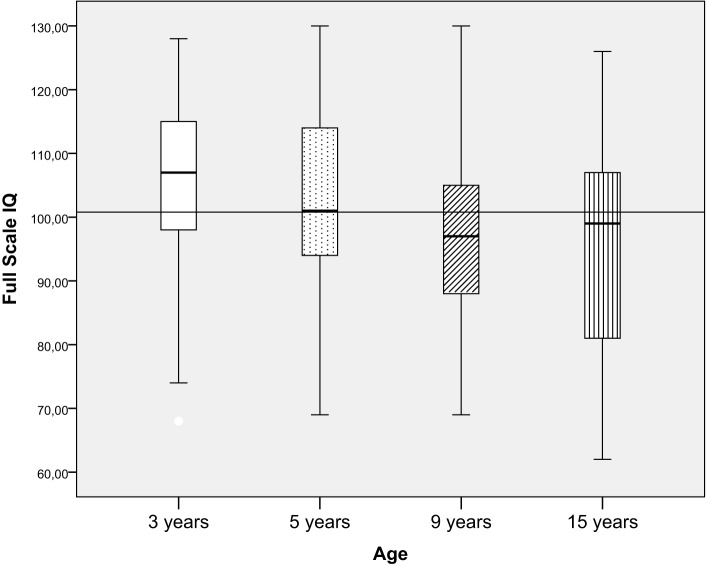
**FSIQ in relation to age**.

**Figure 5:**
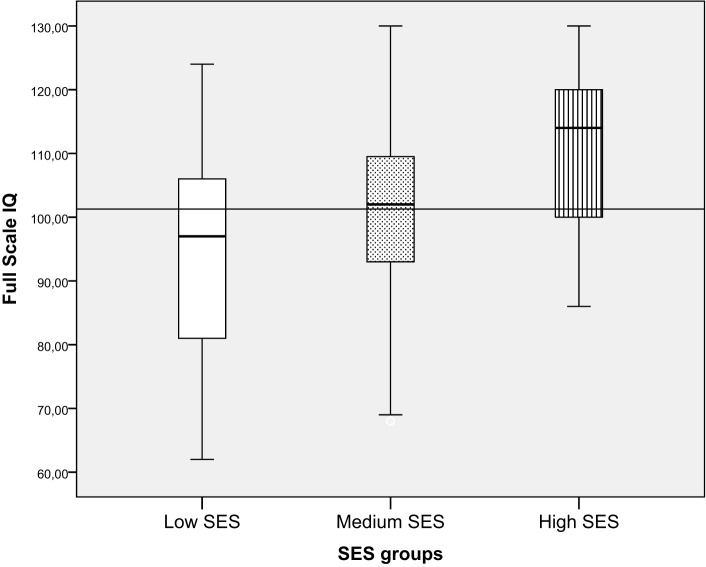
**FSIQ in relation to SES**.

**Figure 6:**
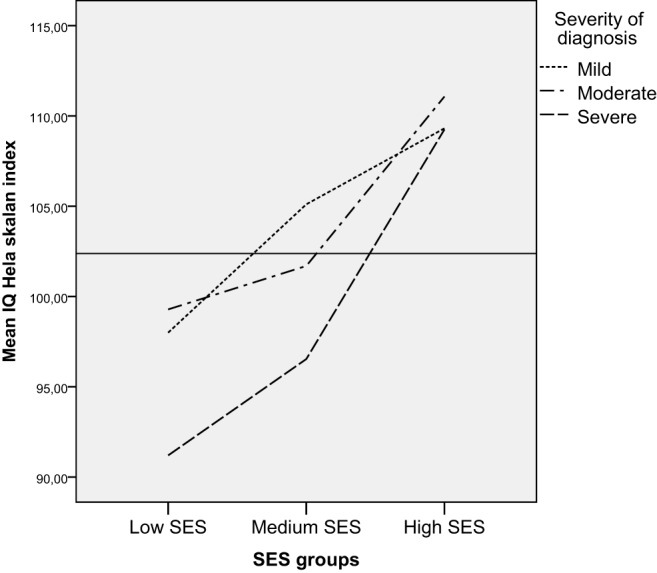
**FSIQ in relation to SES and severity of the cardiac diagnosis**.

For the 228 children, the total mean score for FSIQ was 100.8 (SD = 14.5), displayed in Figures 3–6 with a horizontal line. No significant differences were found between boys and girls. Figure 2 shows the distribution of children performing within the normal range and ±1 and ±2 SD compared to norms.

In the following sections, FSIQ will be presented in relation to severity of diagnosis, age, and SES. We begin each section by presenting FSIQ as a continuous variable, followed by presenting FSIQ as a categorical variable [i.e., the proportion of children performing within the normal range, ±1 SD, +1 SD (no child over +2 SD), and −1 and −2 SD in relation to severity of diagnosis, age, and SES]. See Table 2 for descriptive data.

**Table 2:** **FSIQ mean and SD by severity of cardiac diagnosis, and age or SES**.

	Mild CHD		Moderate CHD		Severe CHD		Total	
	Mean	SD	Mean	SD	Mean	SD	Mean	SD
**AGE**								
3 years (*n* = 65)	109.5	11.0	108.9	12.4	102.0	14.8	105.6	13.6
5 years (*n* = 53)	107.9	12.1	104.4	12.5	95.7	15.2	102.4	14.2
9 years (*n* = 61)	101.1	12.5	99.1	12.6	93.3	13.9	98.0^a^	13.2
15 years (*n* = 49)	95.8	15.6	100.8	11.3	90.9	20.6	96.1^a^	15.9
Total (*n* = 228)	103.6	13.6	103.1	12.6	95.8	16.0	100.8	14.5
**SES**								
Low (*n* = 41)	98.0	15.5	99.3	17.7	91.2	16.5	94.9^c^	16.4
Medium (*n* = 136)	105.1	11.8	101.7	11.6	96.5	15.0	101.0	13.2
High (*n* = 34)	109.3	12.9	111.1	12.1	109.3	16.8	110.0^d^	12.6
Total (*n* = 211)	104.6	13.2	103.2	12.8	95.5^b^	15.9	101.3	14.4

*^a^Intellectual functioning was significantly lower for the 9- and 15-year olds compared to the 3-year-olds [F (3, 227) = 5.3, p = 0.001, $\eta_{\text{p}}^{2}=0.07$]*.

*^b^Children in the severe CHD group had significantly lower FSIQ than children in the mild CHD group and the moderate CHD group [F (2, 227) = 7.3, p = 0.001, $\eta_{\text{p}}^{2}=0.06$]*.

*^c^Children from families with low SES had significantly lower intellectual functioning compared with both the medium SES and the high SES group (mean = 110.0, SD = 12.6)*.

*^d^The high SES group had significantly higher FSIQ than the medium SES group [F (2, 210) = 11.3, p = 0.000, $\eta_{\text{p}}^{2}=0.10$]*.

### Intellectual Functioning in Relation to Severity of Diagnosis

Children in the severe CHD group had significantly lower FSIQ (mean = 95.8, SD = 16.0) than children in the mild CHD group (mean = 103.6, SD = 13.6) and the moderate CHD group (mean = 103.2, SD = 12.6) [*F* (2, 227) = 7.3, *p* = 0.001, $\eta_{\text{p}}^{2}=0.06$], see Figure 3. The effect size was small for distribution of children from the different severity groups performing within the normal range and ±1–2 SD. More children with severe CHD had FSIQ below −1 SD (32.5%) than children with milder forms of CHD (9.3%) [λ^2^ (df 6) = 23.7 *p* < 0.001]. Table 3.

**Table 3:** **Proportion of children with low, average, or high intellectual functioning in relation to severity of diagnosis**.

	Mild CHD *n* (%)	Moderate CHD *n* (%)	Severe CHD *n* (%)	Total *n* (%)
Low FSIQ (−1 and −2 SD)	7 (9.6)	7 (9.0)	25 (32.5)^a^	39 (17.1)
Average FSIQ (within ±1 SD)	48 (65.8)	56 (72.0)	44 (57.1)	148 (64.9)
High FSIQ (+1 SD IQ)	18 (24.7)	15 (9.2)	8 (10.4)	41 (18.0)
Total *n* (%)	73	78	77	228 (100)

*^a^More children with severe CHD had FSIQ below −1 SD (32.5%) than children with milder forms of CHD (9.3%) [λ^2^ (df 6) = 23.7 p < 0.001]*.

### Intellectual Functioning in Relation to Age

Intellectual functioning was significantly lower for the 9-year olds (mean = 98.0, SD = 13.1) and 15-year olds (mean = 96.1, SD = 15.9) compared to the 3-year olds (mean = 105.6, SD = 13.6) [*F* (3, 227) = 5.3, *p* = 0.001, $\eta_{\text{p}}^{2}=0.07$], and the effect size was small. However, when children were divided into categorical IQ-groups (low, average, and high intellectual functioning), there were no significant differences in the proportion of children in the groups in relation to age (Figure 4; Table 2).

### Intellectual Functioning in Relation to SES

Children from families with low SES had significantly lower intellectual functioning (mean = 94.9, SD = 16.4) compared with both the medium SES (mean = 101.0, SD = 13.2) and the high SES group (mean = 110.0, SD = 12.6). The high SES group also had significantly higher FSIQ than the medium SES group [*F* (2, 210) = 11.3, *p* = 0.000, $\eta_{\text{p}}^{2}=0.10$]. The effect size was of medium size. When children were divided into categorical IQ-groups (low, average, and high intellectual functioning), a higher proportion of children from families with low SES had FSIQ below −1 SD (34.1%) compared to children from families with average (14.7%) or high SES (2.9%) [λ^2^ (df 6) = 33.0 *p* < 0.01] (Figure 5; Table 4).

**Table 4:** **Proportion of children with low, average, or high intellectual functioning in relation to SES**.

	Low SES *n* (%) mean and SD	Medium SES *n* (%)	High SES *n* (%)	Total *n* (%)
Low FSIQ (−1 and −2 SD)	14 (34.1)^a^	20 (14.7)	1 (2.9)	35 (16.5)
Average FSIQ (within ±1 SD)	22 (53.7)	97 (71.3)	17 (50.0)	136 (64.5)
High FSIQ (+1 SD IQ)	5 (12.2)	19 (14.0)	16 (47.1)	40 (19.0)
Total *n* (%)	41 (19.4)	136 (64.5)	34 (16.1)	211 (100)

*^a^A higher proportion of children from families with low SES had FSIQ below −1 SD compared to children from families with average or high SES [λ^2^ (df 6) = 33.0 p < 0.01]*.

### Interaction Effects

We found no interaction effect between severity of diagnosis and SES for FSIQ. Using ANOVA to control for the effect of SES on FSIQ, we found that the effect size for severity of diagnosis on FSIQ decreased from.06 to.05 after controlling for SES, but the differences in FSIQ between the severity groups remained significant. Both SES and severity of CHD diagnosis had significant main effects on FSIQ. When children were divided into categorical IQ-groups (low, average, and high intellectual functioning), a larger proportion of children both diagnosed with severe CHD and living in families with low SES performed in the low range of intellectual functioning more often than children diagnosed with severe CHD living in families with medium and high SES (Figure 6) [Pearson chi-square (df 6) = 25.6, *p* < 0.01]. Among those children having low SES and a severe CHD 8 of 20 (40%) had low IQ (−1 or 2 SD); those having medium SES and severe CHD 13 of 43 (30%) had low IQ, and those with high SES severe CHD, 1 (of 4) had low IQ.

## Discussion

In our study of 228 Swedish children with CHD treated with surgery or by catheter technique, the mean score on FSIQ in Wechsler Scales of Intelligence was 100.8 (SD = 14.5). This finding indicates that children with CHD treated with surgery or by catheter interventions as a group performed within the normal range on overall intellectual functioning, a result that contradicts some early studies such as the ones presented in a literature review by Amianto, et al. (8). In this literature review, some studies reported that CHD in children was related to lower intellectual functioning; however, other studies found normal performance in these children not only with respect to general intelligence but also with respect to academic results, learning abilities, and visuospatial abilities (8).

Although 65% of children in the present study performed within the normal range, 17% had scores −1 or −2 SD below the mean, and 18% had scores +1 SD above the mean. When comparing subgroups of children with CHD, some children are clearly more at risk than others in terms of intellectual functioning. As hypothesized, intellectual functioning in children with CHD was related to severity of diagnosis, age, and SES.

In this study, we created similarly sized diagnosis groups with diverse severity levels to avoid focus on specific heart defects, a limitation of many other studies (17, 24–26). The results show that children with severe CHD had significantly lower intellectual functioning than children with mild or moderate CHD. This finding agrees with studies reporting that children with milder forms of CHD, such as ventricular septal defect, present lower incidence of neurodevelopmental problems than children with more severe forms of CHD (6, 35). The fact that children with more severe forms of CHD present higher risk for neurodevelopmental problems suggests that cognitive problems could be related to intraoperative factors and to the surgery procedures themselves (36). Although some studies have evaluated the risks that specific surgery techniques confer on children’s intellectual functioning, no clear relation has been proven (29). However, genetic comorbidities and neurological status before surgery are shown to be significant (37). Brain development during fetal and early postnatal life has shown to be influenced by environmental conditions such as maternal stress, and this psychosocial strain in turn influences intellectual functioning (38, 39). A study measuring brain size in infants with CHD found that although brain size in these children was smaller than in healthy term infants, cerebral grow rates were comparable with the cerebral growth rates of the controls (4), and there were no significant differences in neurodevelopmental outcomes in pre-term-born infants with CHD compared to term-born infants (40, 41). Studies have highlighted the long-term effects of preoperative status (42, 43) and of the surgical procedures, which are determined by the severity of the heart defect (1, 2, 23). In the present study, the SD in the severe group was shown to be wider than in the mild and moderate groups, i.e., the difference in intellectual functioning between the lowest and highest performance in the severe group was larger than in the mild or moderate groups. This finding has also been observed in previous studies (25, 44, 45).

Because using unrestricted or very restricted age groups is a well-known problem in many previous studies (27–29), we created specific comparable age groups. Results showed that children in the older groups (9- and 15-year olds) had significantly lower intellectual functioning compared to the 3-year olds. Three-year olds were assessed with WPPSI–III, and the 9- and 15-year olds were assessed with WISC–IV, a choice that probably influenced the results. Although good correlations have been established between these instruments, the WPPSI–III produces slightly higher scores than the WISC–IV. In addition, as children become older and progress through school, the demands on their intellectual functioning increase. Even though intellectual functioning is one of the best predictors of school performance, there are specific cognitive functions that influence learning (e.g., attention and memory). These specific cognitive factors are not targeted in the FSIQ of the Wechsler scales. Deficits in specific cognitive factors were shown in one study of children with CHD tested at age 5 and 10 years; despite stable intelligence scores, the risk for cognitive deficits increased with age (42). Difficulties with specific cognitive functions – e.g., attention, working memory, and processing speed – may not impact general performance in a test situation such as the Wechsler scales of intelligence but may become evident in everyday situations as demands in school increase with age. The clinical experience is that children with CHD more often require special education and learning interventions when they are older. Future research should target specific cognitive domains such as attention, working memory, and executive function in children with CHD in relation to FSIQ and school performance.

Our results showed a significant relation between SES and intellectual functioning in children with CHD. Children from families with low SES had significantly lower intellectual functioning compared with both the medium SES and the high SES groups. This goes in line with studies carried out in healthy populations in which intellectual functioning is believed to be determined by socioeconomic status (21, 46). Results in our study showed that the high SES group also had significantly higher FSIQ compared to the medium SES group. Larger proportion of the children from families with low SES had FSIQ below −1 SD compared to children from families with average or high SES. Yet, it is important to interpret these results cautiously since our sample had very small subgroups. This finding, however, corresponds with previous studies showing that parental education (28), environmental processes (15, 47), and parental stress (48) are related to intellectual functioning in children with CHD (14). Furthermore, studies reporting results of interventions aimed to promote intellectual functioning, development, emotional adjustment, and resilience in children with CHD have shown that reduced levels of anxiety in mothers, good mental health in parents, and good family functioning are significant advantages not only for intellectual functioning (and fewer missed school days) but also for self-perceived health (49, 50).

In the ANOVA, no interaction effect was found between severity of diagnosis, age, and SES, probably because of the relatively small sample size. However, when using non-parametric test, children simultaneously exposed to severe CHD diagnosis and to low SES were found to more often perform in the low range of intellectual functioning. This finding also corresponds with previous studies showing that children with severe heart defects and lower SES are at greater risk for problems related to intellectual functioning (14, 51).

In sum, children with CHD as a group performed well on FSIQ, although we identified severity of the heart diagnosis and SES as factors related to increased risk for lower FSIQ in children with CHD. We believe that providing parents with specific and accurate information on the risks of lower intellectual functioning, supporting schools with psychoeducational advice, and introducing follow-up and intervention programs for families as early as possible are important steps to improving the outcomes for children with CHD. Therefore, children with severe CHD and children from low SES families should be assessed for further interventions and included in follow-up and intervention programs.

### Limitations

This study is limited by the absence of a control group. Although believed to be reliable, norm data have limitations since we do not know if the groups are comparable on important background variables. Another limitation of this study is the use of only one dependent variable, FSIQ. Although intellectual functioning is one of the best predictors of school performance, it does not fully capture children’s learning problems or behavioral difficulties. Therefore, we believe that future studies should address specific cognitive functions (e.g., executive function, attention, and memory functions) as often these functions are impaired in children seeking help for school problems in this group. Analysis of these functions together with FSIQ could give a more complete understanding of the problems presented in children with CHD.

The response rate in the severe group was much higher than the response rate in the mild group. We do not have background data on the non-responders, and there is a risk of response-bias. It is common that parents with higher education more often agree to participate in scientific studies compared to parents with lower education. It might also be the case that parents of children with more difficulties at school were more inclined to participate because they wanted a cognitive evaluation of their child and more support from the school. We know that FSIQ in our mild CHD group was normally distributed, and the FSIQ scores in the severe CHD group were skewed with lower values on FSIQ. Looking closer at the severity of diagnosis- and SES-relation, we could see that 23% of children in the mild group had high SES while only 7% of children in the severe group had high SES. FSIQ in the mild group could be systematically higher because parents with high IQ and higher education more often agreed to participate or FSIQ in the mild could be lower than expected because parents who were worried about their child more often agreed to participate. The moderate and severe groups’ participation level was much higher, and the risk for systematic response bias lower, although it is possible that the most fragile families, such as families with very ill children and very low socioeconomic status, did not participate. More studies on larger samples are needed to confirm the results of the present study.

Because this is a cross-sectional study, effects should be interpreted with caution. The differences between age groups could be due to many factors. Factors such as older and less effective surgical techniques used in the older children and the fact that different tests were used in younger and in older children contribute to the uncertainty of the results. The WIPPSI is usually considered to produce higher FSIQ scores than the WISC, which may account for the higher scores among the 3-year olds. Also, cognitive assessment in younger children is more problematic and unstable than in older ones (52). Therefore, longitudinal studies of children with CHD are needed to develop an accurate picture of cognitive development.

## Author Contributions

CR has substantially contributed to the design of the work, the acquisition, analysis, and interpretation of the data. JS has substantially contributed to the design of the work, retrieval of patients, classification of severity of diagnosis, and interpretation of the data. MT has substantially contributed to the acquisition, analysis, and interpretation of the data. MB has substantially contributed to the design of the work, analysis, and interpretation of the data. All authors – CR, JS, MT, and MB – have revised the work critically, approved of the final version, and agreed to it.

## Conflict of Interest Statement

The authors declare that the research was conducted in the absence of any commercial or financial relationships that could be construed as a potential conflict of interest.

## Footnotes

^1^Adams J, Weakliem DL. August B. Hollingshead’s “four factor index of social status”: from unpublished paper to citation classic. *Yale J Sociol* (2011) 8:11–20.
